# Characteristics and outcomes of acute respiratory distress syndrome related to COVID-19 in Belgian and French intensive care units according to antiviral strategies: the COVADIS multicentre observational study

**DOI:** 10.1186/s13613-020-00751-y

**Published:** 2020-10-06

**Authors:** David Grimaldi, Nadia Aissaoui, Gauthier Blonz, Giuseppe Carbutti, Romain Courcelle, Stephane Gaudry, Aurelie Gaultier, Alain D’hondt, Julien Higny, Geoffrey Horlait, Sami Hraiech, Laurent Lefebvre, Francois Lejeune, Andre Ly, Michael Piagnerelli, Bertrand Sauneuf, Nicolas Serck, Thibaud Soumagne, Piotr Szychowiak, Julien Textoris, Benoit Vandenbunder, Christophe Vinsonneau, Jean- Baptiste Lascarrou, for the COVADIS study group, Patrick Biston, Gwenhael Colin, Oriane de Maere, Nathan Ebstein, Stephan Ehrmann, Frederic Foret, Lionel Haentjens, Thibault Helbert, Jean-Baptiste Mesland, Celine Monard, Nicolas Mongardon, Gregoire Ottavy, Thomas Pasau, Gael Piton, Ester Ponzetto, Caroline Sejourne, Morgane Snacken, Xavier Souloy, Aude Sylvestre, Nicolas Tartrat, Cedric Vanbrussel

**Affiliations:** 1 Soins IntensifsHôpital Erasme, ULB, Route de Lennik 808, 1070 Bruxelles, Belgium; 2 Medecine Intensive Reanimation, Hôpital Européen Georges Pompidou, Paris centre U 970 PARCC, Paris, France; 3 District Hospital Center, Boulevard Stephane Moreau, Medecine Intensive Reanimation, 85000 La Roche Sur Yon, France; 4 Unité de Soins Intensifs, CHR Mons-Hainaut, Mons, Belgium; 5 Unité de Soins Intensifs, Centres Hospitaliers de Jolimont, La Louvière, Belgium; 6 Réanimation médico-Chirurgicale CHU Avicennes, Université Sorbonne Paris Nord, Bobigny, France; 7 Plateforme de Méthodologie Et Biostatistique, CHU Nantes, 1 Places Alexis Ricordeau, 44093 Nantes Cedex 9, France; 8 Unité de Soins Intensifs, CHU Ambroise Paré, Mons, Belgium; 9 Unité de Soins Intensifs, CHU Dinant Godinne, site Dinant, Dinant, Belgium; 10 Unité de Soins Intensifs, CHU Dinant Godinne, site Godinne, Godinne, Belgium; 11 Médecine Intensive Réanimation, Assistance Publique - Hôpitaux de Marseille, Hôpital Nord, 13015 Marseille, France; 12 Centre D’Etudes Et de Recherches Sur Les Services de Santé Et qualité de Vie EA 3279, Aix- Faculté de médecine, Marseille Université, 13005 Marseille, France; 13 Réanimation Polyvalente Centre Hospitalier du Pays D’Aix, Aix en Provence, France; 14 Unité de Soins Intensifs, Clinique Notre Dame de Grâce, Gosselies, Belgium; 15 Service d’anesthésie-réanimation chirurgicale Unité de réanimation chirurgicale polyvalente, Hôpitaux Universitaires Henri Mondor, Créteil, France; 16 Intensive Care. CHU-Charleroi, Marie Curie, Université Libre de Bruxelles, 140, chaussée de Bruxelles, 6042 Charleroi, Belgium; 17 Réanimation - Médecine Intensive, Centre Hospitalier Public du Cotentin, BP208, 50102 Cherbourg-en-Cotentin, France; 18 Unité de soins intensifs, Clinique Saint Pierre, Ottignies, Belgium; 19 Médecine Intensive Réanimation, CHU Besançon, 3 Boulevard FLEMING, 25030 Besançon, France; 20 Médecine Intensive Réanimation, CHRU Tours, Tours, France; 21 INSERM CIC 1415, CHRU Tours, Tours, France; 22 CRICS-TriggerSEP research network, Tours, France; 23 Service de réanimation, Hospices Civils de Lyon, 5 Place D’Arsonval, Lyon, France; 24 Laboratoire Commun de Recherche bioMérieux-Hospices Civils de Lyon-Université de Lyon 1, EA7426 PI3 Lyon, France; 25 Groupe des anesthésistes réanimateurs, Hôpital Privé d’Antony, Antony, France; 26 Service de Médecine Intensive Réanimation Unité de Sevrage Ventilatoire et Réhabilitation Centre Hospitalier de BETHUNE, 27 Rue Delbecque, 62660 Beuvry, France; 27 Medecine Intensive Reanimation, CHU Nantes, 30 Boulevard Jean Monnet, 44093 Nantes Cedex 9, France

**Keywords:** Renal replacement therapy, Remdesivir, Lopinavir, Ritonavir, Acute kidney injury, Hydroxychloroquine

## Abstract

**Background:**

Limited data are available regarding antiviral therapy efficacy in most severe patients under mechanical ventilation for Covid-19-related acute respiratory distress syndrome (ARDS).

**Methods:**

Comparison of antiviral strategies (none, hydroxychloroquine (OHQ), lopinavir/ritonavir (L/R), others (combination or remdesivir) in an observational multicentre cohort of patients with moderate-to-severe Covid-19 ARDS. The primary endpoint was the number of day 28 ventilator-free days (VFD). Patients who died before d28 were considered as having 0 VFD. The variable was dichotomized into “patients still ventilated or dead at day 28” versus “patients weaned and alive at day 28”.

**Results:**

We analyzed 415 patients (85 treated with standard of care (SOC), 57 with L/R, 220 with OHQ, and 53 others). The median number of d28-VFD was 0 (IQR 0–13) and differed between groups (*P* = 0.03), SOC patients having the highest d28-VFD. After adjustment for age, sex, Charlson Comorbidity Index, PaO_2_/FiO_2_ ratio and plateau pressure and accounting for center effect with a generalized linear mixed model, none of the antiviral strategies increased the chance of being alive and weaned from MV at day 28 compared to the SOC strategy (OR 0.48 CI95% (0.18–1.25); OR 0.96 (0.47–2.02) and OR 1.43 (0.53–4.04) for L/R, OHQ and other treatments, respectively). Acute kidney injury during ICU stay was frequent (55%); its incidence was higher in patients receiving lopinavir (66 vs 53%, *P* = 0.03). After adjustment for age, sex, BMI, chronic hypertension and chronic renal disease, the use of L/R was associated with an increased risk of renal replacement therapy (RRT). (OR 2.52 CI95% 1.16–5.59).

**Conclusion:**

In this multicentre observational study of moderate-to-severe Covid-19 ARDS patients, we did not observe any benefit among patients treated with OHQ or L/R compared with SOC. The use of L/R treatment was associated with an increased need for RRT.

*Take home message *Neither hydroxychloroquine nor lopinavir/ritonavir as COVID-19 antiviral treatment is associated with higher ventilator-free days at day 28 when compared with standard of care (no antiviral treatment) in ICU patients under invasive mechanical ventilation. Lopinavir/ritonavir is associated with an increased risk of renal replacement therapy requirement.

*Tweet* COVID-19: Insights from ARDS cohort: no signal of efficacy of any antiviral drugs. Lopinavir/ritonavir may be associated with need for RRT

## Introduction

Since December 2019, Coronavirus Disease 2019 (COVID-19) caused by severe acute respiratory syndrome coronavirus 2 (SARS-CoV-2) emerged in Wuhan, China, and rapidly spread throughout China, Asia and the world. COVID-19 can have different clinical presentations, but respiratory symptoms predominate especially in patients requiring intensive care unit (ICU) admission [1]. Respiratory symptoms mainly affect adults over 50 years of age, predominantly males with cardiovascular comorbidities [1, 2]. The respiratory features are characterized by severe hypoxemia, radiological ground glass opacities and especially crazy paving. Its evolution is prolonged with an aggravation phase 7–10 days after symptoms onset [3] leading to death between 3% of patients requiring conventional hospitalization [4] to 60% of patients requiring ICU [5].

Despite expert recommendations that were implemented quickly [6], the management was not based on high levels of evidence during the West European first wave (March–April 2020). The treatments applied varied from one country to another and from one center to another. Frantic rush towards the most efficient antiviral therapy has been ongoing since the epidemic started. First candidates were already developed molecules that demonstrated in vitro effects, mainly remdesivir [7], lopinavir/ritonavir [8] and hydroxychloroquine [9]. The last two were already on the market with an acceptable safety profile. However, they have not been used widely in critically ill patients and some potential unknown side effects may exist. Even though some randomized clinical trials have now been published for remdesivir [10], or for lopinavir/ritonavir alone [6] or in association [11], most of the patients in these trials were managed in conventional wards with few patients requiring mechanical ventilation and ICU.

We conducted an observational international multicentre study assessing patients suffering from moderate-to-severe COVID-19-related ARDS to detect a possible signal of efficacy or deleterious effects of these antiviral therapies. Herein, we report the outcomes of these COVID-19 patients admitted to ICU and requiring invasive mechanical ventilation according to the chosen therapeutic strategy among several antivirals or standard of care.

We hypothesized that antiviral therapies may decrease the length of invasive ventilation, assessed by the number of ventilator-free days (VFD) at day 28.

## Patients and methods

This study complied with the STROBE guidelines [12].

### Study design and setting

The COVADIS project was observational and included 21 ICUs in France (*n* = 12) and Belgium (*n* = 9).

### Patient selection

Inclusion criteria were:

- Age older than 18 years,
- Moderate-to-severe ARDS according to the Berlin definition [13] (PaO_2_/FiO_2_ ratio < 200 mmHg with a PEEP of at least 5 mmHg receiving invasive ventilation),
- Positive SARS-CoV-2 reverse transcriptase polymerase chain reaction (PCR) regardless of the sampling site (patient with negative PCR but chest CT scan with abnormalities such as crazy paving were not included).

Non-inclusion criteria were:

- Cardiac arrest before ICU admission,
- Extra corporeal membrane oxygenation (ECMO) requirement within first 24 h of ICU length,
- Gold class 3 or 4 chronic obstructive pulmonary disease, or home oxygen requirement.

### Data collection

For this observational multicenter study, all consecutive COVID-19 patients admitted to the participating centers between March 10, 2020 and April 15, 2020 were screened. Patients fulfilling inclusion and non-inclusion criteria were included. Each local investigator filled out an eCRF to collect data (Castor EDC, Amsterdam, The Netherlands). We recorded demographic data, known medical history and co-morbidities using the Charlson score [14], as well as history of chronic hypertension. We collected data regarding medical management during ICU stay including settings of mechanical ventilation after intubation, duration of mechanical ventilation, use of advanced therapies for acute respiratory failure (neuromuscular blocking agents, inhaled pulmonary vasodilators, prone-positioning, and extracorporeal membrane oxygenation), antiviral therapies and immunomodulatory agents (i.e., interleukin-6-receptor antagonists and corticosteroids) with time from onset of symptoms to initiation, occurrence of acute kidney injury (AKI), acute cardiac injury (defined as a rise in troponin level over 10 times the normal threshold or as the need for inotropes), and occurrence of pulmonary embolism and deep venous thrombosis.

## Definitions

Patients receiving antiviral therapy were defined as patients having received at least one complete day of respective antiviral therapy. As many candidates for specific antiviral treatment were under evaluation, our variable of interest was the use of antiviral treatment according to one of the pre-specified following category: none (standard of care), lopinavir/ritonavir (AbbVie, Rungis, France), hydroxychloroquine (Sanofi, Gentilly, France), and others (more than one antiviral treatment or remdesivir (Gilead, Foster City, USA) as it was not commercially available). More details regarding treatment doses and duration are provided in the Additional file 1: Table S1.

## Outcomes

### Primary objective and endpoint

The primary objective was to assess the outcome of COVID-19 patients requiring invasive mechanical ventilation and ICU according to antiviral strategies.

The primary endpoint was the number of ventilator-free days at day 28.

Considering the absence of efficacy data regarding antiviral treatments in the setting of critically ill patients requiring invasive mechanical ventilation, we decided to choose a composite outcome which included death and length of mechanical ventilation: number of ventilator-free days (VFD) at day 28 [15]. VFD at day 28 was determined as follows:

- VFDs = 0 if subject died within 28 days of mechanical ventilation,
- VFDs = 28 − *x* if successfully liberated from ventilation *x* days after initiation,
- VFDs = 0 if the subject was mechanically ventilated for > 28 days.

The variable was dichotomized into “patients still ventilated or dead at day 28” (VFD = 0) vs “patients weaned and alive at day 28” (VFD > 0).

### Secondary objectives and endpoints

The secondary objectives were to assess if the use of antiviral therapy improved the outcomes in terms of short-term survival, duration of mechanical ventilation, cardiac injury, acute kidney injury, requirement of renal replacement therapy and occurrence of thromboembolic disease.

The secondary endpoints were:

- Survival at day 14,
- Ventilator mode at day 14 according to predefined 4 categories: patient under volume/pressure assisted controlled or ECMO, pressure support mode, spontaneous breathing while extubated, death,
- Survival at day 28,
- Occurrence of AKI during the first 28 days after intubation, defined by a rise in serum creatinine of at least 50% as per KDIGO stage 1 definition [16], and classified as none, present without need for renal replacement therapy (RRT), present with need for RRT,
- Peak of creatinine during the first 28 days following intubation,
- Acute cardiac injury (defined as plasmatic troponin level upper than 10 times upper normal range or need for inotropes (dobutamine, epinephrine, milrinone, and/or levosimendan),
- Deep venous thrombosis and pulmonary embolism,
- Survival at ICU discharge.

### Statistical analysis

Discrete data were described by their frequency expressed as a percentage. Numerical data were described by the mean and standard deviation.

Discrete data were compared using a Chi-square test or Fisher’s exact test, as appropriate. Continuous normally distributed data were compared by ANOVA and continuous non-normal data by Kruskal–Wallis as appropriate.

A pre-planned multivariate analysis was performed to identify factors associated with day 28 VFD. We included in the model all the variables associated with day 28 VFD in univariate analysis with a *P* value < 0.10 and variables with known prognostic value in ARDS. We forced in the model the type of antiviral strategy as categorical variable. Given (1) the non-normal distribution of day 28 VFD and (2) the median value at 0, according to pre-specified rules in the protocol, we discriminated the day 28 VFD variables in having at least one day of VFD or not, ie. being extubated and alive at day 28 or not. We performed then a generalized linear mixed model with center as random effect. SOC group was defined as the reference group for the antiviral strategy variable. Step-by-step backward selection was applied. The final model included only variables with *P* value below 0.05. Hosmer–Lemeshow test and visual inspection of residues were used to ensure the quality of the regression.

On reviewer demand and considering unforeseen high length-of-stay in ICU for COVID-19 patients when the study was designed, we analyzed the factors associated with survival at ICU discharge with univariate analysis and using a post hoc generalized linear mixed model as described above.

Post hoc generalized linear mixed models on the factors associated with AKI and with the need for RRT were performed adjusting antiviral molecule (administered in mono- or combined combination therapy) associated with AKI in univariate analysis (*P* < 0.10) with age, sex, BMI, chronic hypertension and moderate-to-severe chronic renal failure.

No imputation strategy was used for missing data. *P* value < 0.05 was considered significant.

All analyses were performed using Stata (version 16, StataCorp, College Station, TX, USA).

### Sample size

According to the paucity of data regarding the efficacy of antiviral treatments, we planned to include at least 250 patients to allow comparison of each antiviral treatment candidate.

### Role of the funding source

This study was not funded by any sources.

## Results

### Baseline characteristics (Table 1)

**Table 1:** Patients’ characteristics according to antiviral strategy

Total	No antiviral	Lopinavir ritonavir	Hydroxychloroquine	Other^a^	*P *value^b^
*N* = 415	*N* = 85	*N* = 57	*N* = 220	*N* = 53	
France (vs Belgium), *n* (%)	77 (91)	56 (98)	96 (43.6)	15 (28)	< 0.001
Age, mean ± SD	63 ± 11	63 ± 12	64 ± 10	62 ± 10	0.54
Gender, male, *n* (%)	64 (75)	46 (80)	169 (77)	42 (79)	0.87
Body mass index, mean ± SD N = 84/57/215/53	30 ± 5	29 ± 5	30 ± 5	29 ± 5	0.47
History of chronic hypertension, *n* (%)	51 (60)	23 (40)	131 (59)	30 (57)	0.06
Charlson Comorbidity Index	1 (0–2)	1 (0–2)	1 (0–2)	0 (0–1)	0.02
0	33 (39)	17 (30)	88 (40)	33 (62)	0.005
1	16 (19)	19 (33)	65 (29)	8 (15)	
2 or more	36 (42)	21 (37)	67 (31)	12 (23)	
Duration between symptoms onset and initiation, median [IQR]	NA	8 [7–10]	7 [5–10]	8 [6–10]	0.18
*N* = *-/49/192/46*					
Macrolides, *n* (%)	34 (40)	47 (82)	158 (72)	21 (40)	< 0.001
Including azithromycin, *N* = 260 (%)	6 (18)	7 (15)	105 (67)	7 (33)	< 0.001
Co-infection, *n* (%)	3 (4)	2 (4)	36 (16)	8 (15)	0.003
Tidal volume, (mL/kg of IBW), mean ± SD	6.2 ± 0.7	6.0 ± 0.5	6.4 ± 1	6.0 ± 0.7	0.004
*N* = 83/57/214/53					
Total PEEP (cmH_2_O), mean ± SD	11 ± 3	11 ± 3	12 ± 3	12 ± 3	0.11
Plateau pressure (cmH_2_O), mean ± SD	23 ± 4	23 ± 3	24 ± 4	22 ± 4	0.004
*N* = 71/45/202/49					
P/F ratio, mean ± SD	132 ± 39	143 ± 54	124 ± 54	117 ± 44	0.02
Driving pressure, mean ± SD	13 ± 4	11 ± 4	12 ± 4	10 ± 3	0.001
*N* = 71/45/202/49					
Neuromuscular blockade, *n* (%)	78 (92)	50 (88)	178 (81)	44 (83)	0.11
Inhaled nitric oxide, *n* (%)	6 (7)	4 (7)	37 (17)	4 (8)	0.03
Prone position, *n* (%)	62 (73)	39 (68)	189 (86)	43 (81)	0.006
Corticosteroids, *n* (%)	10 (15)	10 (18)	52 (24)	13 (25)	0.41
*N* = 393^c^					
Inhibitor of Il6, *n* (%)	1 (1)	0	9 (4)	0	0.12

^a^Including OHQ + L/R *N* = 32; OHQ + REM *N* = 10; REM *N* = 11

^b^*P* value was calculated using Chi-2, ANOVA, Kruskal–Wallis as appropriate

^c^Some patients were included in a double blind RCT steroids vs placebo (NCT02517489)

*ARDS* acute respiratory distress syndrome, *IBW* ideal body weight, *P/F ratio* PaO_2_/FiO_2_ ratio, *OHQ* hydroxychloroquine, *L/R* lopinavir/ritonavir, *REM* remdesivir

From March 10, 2020 to April 15, 2020, 415 patients were included in the study (Additional file 1: Figure S1). They were mostly male (*n* = 321, 77%) with mean body mass index (30 ± 5 kg/m^2^) and suffered from minimal comorbidities previous to COVID-19 as highlighted by Charlson score (1 [0–2]). The most frequent comorbidity was chronic hypertension (*n* = 235 patients; 57%). Patients’ demographic characteristics were similar across different treatment groups (Table 1).

### Antiviral strategies

The most frequently administered antiviral treatment was hydroxychloroquine monotherapy (OHQ) (*n* = 220; 53%), followed by lopinavir/ritonavir monotherapy (L/R) (*n* = 57; 14%). Other therapies (OTH) (combination or REM) were used in 53 patients (11 REM, 32 OHQ + L/R, 10 OHQ + REM). Eighty-five patients did not receive any supposedly active treatment against SARS-CoV2 (Standard of care, SOC). Of note, antiviral treatments were initiated after 8 [5–10] days of symptoms onset without difference between L/R, OHQ, or OTH groups (*P* = 0.18).

### Primary objectives (Table 2 and Fig. 1)

**Table 2:** Outcome according to antiviral treatment

	No antiviral	Lopinavir ritonavir	Hydroxychloroquine	Other^a^	*P*^b^ value
	*N* = 85	*N = *57	*N* = 220	*N* = 53	
Ventilatory mode at day 14, *n* (%)					
Death					0.32
Controlled mode or under VV-	15 (18)	12 (21)	58 (27)	9 (17)	
ECMO	27 (32)	20 (35)	72 (33)	22 (42)	
Pressure support	16 (19)	12 (21)	45 (21)	14 (26)	
Extubated	27 (32)	13 (23)	44 (20)	8 (15)	
Alive at day 28, *n* (%)	62 (73)	35 (61)	140 (64)	38 (72)	0.30
VFD at day 28, median [IQR]	5 [0–15]	0 [0–11]	0 [0–12]	0 [0–9]	0.03
*N* = 80/49/197/7/43					
Acute kidney injury, *n* (%)					
No	38 (45)	17 (30)	106 (48)	22 (42)	0.002
Yes without RRT	33 (39)	18 (32)	72 (33)	26 (49)	
Yes with RRT	14 (17)	22 (39)	41 (19)	5 (9)	
Peak of creatinine, median [IQR]	115 [84–209]	198 [95–432]	122 [80–316]	145 [88–245]	0.05
*N* = 84/52/218/53					
Acute cardiac injury, *n* (%)	8 (9)	6 (10)	37 (17)	4 (8)	0.14
Pulmonary embolism, *n* (%)	11 (13)	6 (11)	32 (15)	10 (19)	0.78
Deep venous thrombosis, *n* (%)	7 (8)	8 (14)	20 (9)	4 (8)	0.62
ICU survival, *n* (%)	56 (67)	27 (51)	123 (57)	34 (64)	0.22
*N* = 406					

^a^ Other including HCQ + L/R *N* = 32; HCQ + REM *N* = 10; REM *N* = 11

^b^ *P* value was calculated using Chi-2, ANOVA, Kruskal–Wallis as appropriate

*RRT* renal replacement therapy, *VFD* ventilator-free days, *VV-ECMO* veno-venous extracorporeal membrane oxygenation

**Fig. 1:**
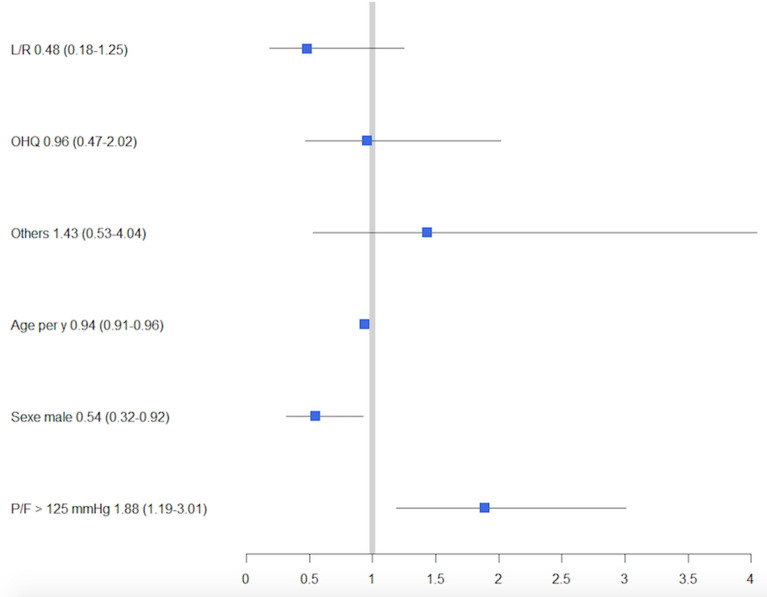
Forest plot of antiviral strategies effect on being alive and extubated at day 28 after adjustment on age, sex, Charlson Comorbidity Index, plateau pressure and P/F ratio. Standard of care served as reference. ORs (CI95%) are indicated after each variable and were obtained through a generalized linear mixed model with center as random effect (*N* = 412). *L/R* lopinavir/ritonavir, *OHQ* hydroxychloroquine, *P/F* PaO_2_/FiO_2_

Overall, the number of day 28 VFD was 0 [0–13] but significantly different across antiviral strategies (*P* = 0.03).

The proportion of patients alive and extubated at day 28 were 53, 32, 39, 49% for SOC, L/R, OHQ and other groups (*P* = 0.04). Compared to the SOC strategy, using an unadjusted analysis, L/R and OHQ treatment were associated with a lower chance to be alive and extubated at day 28 (OR 0.41 CI95% 0.20–0.83 and OR 0.57 CI95% 0.34–0.95), whereas other therapies were associated with a similar chance (OR 0.86 CI95% 0.43–1.70). However, after adjustment on age, sex, Charlson Comorbidity Index, PaO_2_/FiO_2_ ratio and plateau pressure and accounting for the center effect, none of the antiviral strategies was significantly associated with a different chance of being alive and weaned from mechanical ventilation at day 28 compared to the SOC strategy (Fig. 1).

### Secondary objectives (Table 2)

Day 14, day 28 and ICU survival were similar across treatment groups. The post hoc multivariate analysis showed that after adjustment and taking into account the center effect with a generalized linear mixed model, none of the antiviral strategies was significantly associated with a different ICU survival compared to the SOC strategy (Additional file 1: Figure S2). The comparison between ICU survivors and non-survivors is provided in the Additional file 1: Table S2.

At day 14, the distribution of ventilator status was similar across treatment groups, most patients being under controlled mode (Table 2).

At day 28, AKI, pulmonary embolism and cardiac injury were present in 56%, 14% and 13% of the patients, respectively. As shown in Table 2, we observed a difference in AKI incidence between the different groups (*P* = 0.002). The occurrence of RRT at day 28 was more frequent (39%) and the maximal creatinine level was higher in the lopinavir/ritonavir group compared to other groups (17%, 19% and 9% for SOC, OHQ and OTH, respectively).

### Factors associated with AKI occurrence (Table 3, Fig. 2, Additional file 1: Figure S3)

**Table 3:** Associated factors with AKI within 28 days after intubation

*N* = 414	AKI −	AKI +	*P* value^a^
	*N* = 183	*N* = 231	
Age, mean ± SD	61 ± 11	65 ± 10	< 0.001
Gender, men, *n* (%)	133 (73)	187 (81)	0.05
BMI, kg/m^2^, mean ± SD	28.9 ± 5	30.4 ± 5	0.006
Hypertension, *n* (%)	83 (45)	151 (65)	< 0.001
Uncomplicated diabetes mellitus, *n* (%)	34 (19)	46 (20)	0.73
Complicated diabetes mellitus, *n* (%)	7 (4)	27 (12)	0.004
Chronic kidney disease, *n* (%)	5 (3)	28 (12)	< 0.001
Peripheral artery disease, *n* (%)	4 (2)	21 (9)	0.003
History of myocardial infarction, *n* (%)	10 (6)	27 (12)	0.04
Charlson Comorbidity Index, median [IQR]	0 (0–1)	1 (0–2)	< 0.001
= 0	92 (50)	79 (34)	< 0.001
= 1	53 (29)	54 (23)	
≥ 2	38 (21)	98 (42)	
PEEP (cmH_2_O), mean ± SD	11.3 ± 3	11.6 ± 3	0.32
Plateau pressure (cmH_2_O), mean ± SD	23.5 ± 4	23.7 ± 4	0.60
*N* = 159/207			
P/F, mean ± SD	130 ± 54	124 ± 47	0.29
NO, *n* (%)	21 (12)	30 (13)	0.64
Hydroxychloroquine, *n* (%)	124 (68)	137 (59)	0.08
Lopinavir/ritonavir, *n* (%)	30 (16)	59 (26)	0.03
Remdesivir, *n* (%)	9 (5)	12 (5)	0.90
Corticosteroids^b^, *n* (%)	35 (21)	50 (23)	0.61
*N* = 221/171			
Macrolides	118 (65)	141 (61)	0.47
Co-infection	19 (10)	30 (13)	0.42
Day 14 ventilatory mode			< 0.001
Death	21 (12)	73 (32)	
Controlled or VV-ECMO	64 (35)	77 (33)	
Pressure support	40 (22)	47 (20)	
Extubated	58 (32)	34 (15)	
Peak creatinine until day 28, median [IQR]	80 [64–95]	253 [147–436]	< 0.001
*n* = 181/205			
Cardiac injury, *n* (%)	10 (6)	45 (20)	< 0.001
Alive at D28, *n* (%)	145 (79)	129 (56)	< 0.001
Day 28 VFD, median [IQR]	8 (0–16)	0 (0–2)	< 0.001
ICU survival, *n* (%)	136 (75)	104 (46)	< 0.001
*N* = 181/225			

^a^ *P* value was calculated by Fisher exact test, *t* test or Mann–Whitney test as appropriate

^b^ Some patients were included in a RCT steroids vs placebo

*AKI* acute kidney injury was defined as at least a rise of at least 1.5 times in serum creatinine, *BMI* body mass index, *SD* standard deviation, *P/F*: PaO_2_/FiO_2_ ratio, *NO* inhaled nitric oxide, *VV-ECMO* veno-venous extracorporeal mobile assistance, *VFD* ventilator-free days

**Fig. 2:**
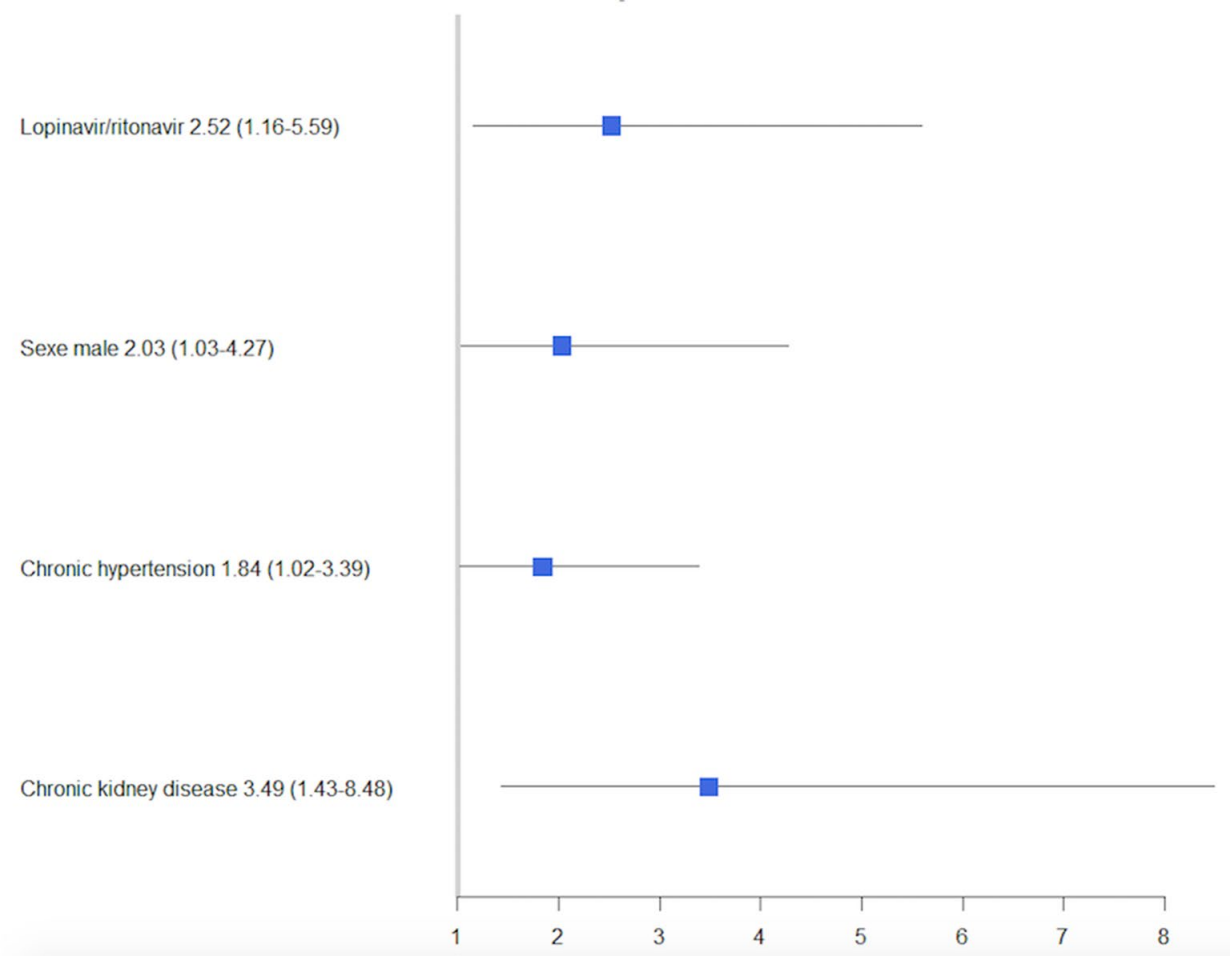
Forest plot of variables associated with need for RRT. OR (CI95%) were obtained through a generalized linear mixed model including lopinavir, hydroxychloroquine age, sex, BMI, chronic hypertension and moderate-to-severe chronic renal failure with center as random effect (*N* = 410). *RRT* renal replacement therapy. Chronic kidney disease: moderate-to-severe chronic renal failure

We compared patients’ characteristics according to the occurrence of AKI (Table 3). Use of L/R, either in mono- or combination therapy (*N* = 89), was significantly more frequent in patients who further developed AKI (26 vs 16%; *P* = 0.03). After adjustment for age, BMI, chronic hypertension and chronic renal failure, and taking into account the center effect, L/R was not significantly associated with AKI (Additional file 1: Figure S3), but was associated with RRT requirement (OR 2.52 CI95% 1.16–5.59, Fig. 2). In this analysis, OHQ treatment was not associated with AKI and RRT.

## Discussion

In this observational, multicentre, binational, study assessing moderate-to-severe ARDS complicating COVID-19 and requiring ICU admission in France and Belgium, we did not observe any benefit of antiviral therapies (L/R, OHQ or combination therapies). There was an association between L/R treatment and the need for RRT. Of note, the low number of patients treated with remdesivir precluded any conclusion regarding this specific therapy.

Our study is in line with previous findings regarding COVID-19 related ARDS in other countries [5, 17]. Patients were mostly overweight males between 50 and 70 years of age, with mostly mild cardiovascular comorbidities. Duration of symptoms before antiviral initiation was in accordance with previous studies: 13 [10–15] days in a study by Cao et al.[8].

Duration of ventilation was high in our cohort, exceeding by far the usual duration in ARDS patients (8 [4–16] days in the LUNG-SAFE cohort [18], with day 28 VFD = 10 [0–22]). However, the day 28 mortality was 34% in our cohort, whereas it was 37% in moderate-to-severe ARDS in the LUNG-SAFE cohort. Of note, ICU mortality of COVID-19 ARDS was higher than day 28 mortality and exceeded 40%. In line with ARDS guidelines [19], physicians in this study set tidal volume near 6 mL/kg of IBW, PEEP at moderate–high level, used prone-positioning liberally and neuromuscular blocking agents [20]. We observed small but significant differences in PEEP level, tidal volume and plateau pressure across patients’ groups (possibly favored by the limited dispersion of the value), but the clinical significance of these differences are uncertain.

Hence, although patients were not randomized, we think that the absence of association between any antiviral treatment and positive outcome (neither at day 28 nor at ICU discharge) deserves consideration. Conversely, we observed a higher day 28 VFD in the group of patients without any antiviral treatment, which raises concerns regarding the safety profile of such antiviral therapies. After adjustment for confounding variables including center effect, none of the strategies appeared significantly superior to SOC.

Since the start of the epidemic, several scientific data emerged on the absence of efficacy of antiviral candidates: no benefit with OHQ [21] and possible higher rate of cardiac events especially if combined with azithromycin [22], no benefit either with L/R in a small randomized controlled trial (RCT) not dedicated to critically ill patients [8]. Concurrently, results of a US trial investigating REM did show a potential benefit on time to recovery but only in patients in conventional wards with a mild disease severity [10]. To date, the only medication with mortality benefit is corticosteroids [23]. As clinical benefit seems unlikely for OHQ and L/R, we believe that only the results of ongoing RCTs will determine if REM has a beneficial effect on outcomes in the subset of patients with ARDS requiring mechanical ventilation. Yet, we agree with current guidelines on antiviral therapy from IDSA [24], NIH [25], and French HCSP [26], to avoid compassionate use of these drugs, following the “first do not harm” rule [27].

Indeed, regarding potential side effects as predefined secondary outcomes, we observed that patients treated by L/R had higher occurrence of AKI and need for renal replacement therapy. The association between L/R treatment and RRT requirement was still present after adjustment for potential cofounders. Several reports in HIV patients indicated that L/R use was associated with an increased risk of chronic kidney disease [28] and acute tubular injury has been described with ritonavir [29]. Renal disease characterized by a proximal tubulopathy has been reported in COVID-19 [30] and has been described as a prognostic factor [31]. We reported a frequent occurrence of AKI in our cohort, in line with US data, with 20% of patients requiring RRT [17]. In our cohort, we observed a strong association between ICU mortality and AKI, although we could not determine if AKI was a marker of a more severe systemic viral sepsis or a causal determinant of survival. This is consistent with a previous report [32]. Interestingly, cardiac injury, but not pulmonary embolism [33], was also associated with increased mortality.

To our best knowledge, no study dedicated to COVID-19 patients requiring ICU analyzed the factors associated with COVID-19 AKI. In our cohort, we observed that, besides treatment with L/R, age, gender, BMI, renal and cardiovascular comorbidities were independently associated with AKI. Higher incidence of AKI may be explained either by a previous nephron loss and/or by a specific susceptibility of these patients to SARS-CoV2 through a greater expression of ACE-2 in podocytes and proximal straight tubule cells [34]. Another possible hypothesis is the occurrence of hypovolemia related to L/R-associated diarrhea and “dry lung” strategy promoted for patients with severe ARDS, although usual hemodynamic monitoring in participating ICUs did not support this hypothesis. Last, recent reports highlighted increased plasma level of lopinavir in patients with COVID-19 compared to HIV-infected patients [35, 36]. Our study was not designed to favor one of these speculative hypotheses. Absence of independent association between lopinavir/ritonavir and AKI after adjustment can be related to overfitting and/or to our sensitive threshold for definition of AKI, highlighted by high rates of AKI as compared to other cohorts with yet similar rate of RRT in groups not receiving L/R [17, 37]. Nevertheless, L/R was independently associated with RRT requirement, which is a worrying finding.

Finally, our observational study has some limits: the patients were not randomized so that we cannot exclude indication bias although collected variables suggested high similarity across treatment strategies. Non-measured confusion biases may exist as well. We did not collect any severity scores, but these scores have been proposed to compare patients with different diseases in the ICU. Of note, we collected Charlson Comorbidity Index, which, associated with gender and age, has been shown to predict mortality with good accuracy [38]. We have also some missing data, which can impact on our results. Given the time constraints during the COVID-19 surge, we strongly limited the number of collected variables so that we were not able to report data regarding organ failures at ICU admission or daily ventilator settings. Our limits are similar to many previous reported studies designed in the same conditions [39]. Our primary outcome might be criticized and might suffer from some drawbacks [40]. Last, some of these patients may have been included in other studies.

## Conclusion

In moderate-to-severe ARDS COVID-19 patients, we did not observe any association between hydroxychloroquine or lopinavir/ritonavir use and ventilator-free days nor ICU mortality, as compared to no antiviral treatment. We observed an independent association between lopinavir/ritonavir and the need for RRT. Our data do not support the use of any of these drugs until results from RCTs dedicated to ICU patients are available.

## Supplementary information

**Additional file 1: Table S1.** Antiviral standard procedure in the participating centers. **Table S2.** Associated factors with ICU survival. **Figure S1.** Flow chart of the study. **Figure S2.** Forest plot of antiviral strategies effect on ICU survival in a mixed multivariate model. **Figure S3.** Forest plot of variables associated with AKI in a mixed multivariate model.

## Data Availability

The database of the study will be freely accessible online within 3 months after publication upon reasonable request to corresponding author.
