# Supplementary material for: Characteristics and outcomes of acute respiratory distress syndrome related to COVID-19 in Belgian and French intensive care units according to antiviral strategies: the COVADIS multicentre observational study
Source: Ann Intensive Care. 2020 Oct 6;10:131. doi: 10.1186/s13613-020-00751-y (PMC7537971; doi:10.1186/s13613-020-00751-y)
Supplement: Supplementary file 1 — Additional file 1: Table S1. Antiviral standard procedure in the participating centers. Table S2. Associated factors with ICU survival. Figure S1. Flow chart of the study. Figure S2. Forest plot of antiviral strategies effect on ICU survival in a mixed multivariate model. Figure S3. Forest plot of variables associated with AKI in a mixed multivariate model. [file 13613_2020_751_MOESM1_ESM.docx]

**Electronic Supplementary Material**

Characteristics and outcomes of Acute Respiratory Distress Syndrome related to COVID-19 in Belgian and French Intensive Care Units according to antiviral strategies.

The COVADIS multicenter observational study.

**Authors**

David Grimaldi^1^, MD; Nadia Aissaoui^2^, MD; Gauthier Blonz^3^, MD; Giuseppe Carbutti^4^, MD; Romain Courcelle^5^, MD; Stephane Gaudry^6^, MD; Aurelie Gaultier^7^, MSc; Alain D’hondt^8^, MD; Julien Higny^9^, MD; Geoffrey Horlait^10^, MD; Sami Hraiech^11,12^, MD; Laurent Lefebvre^13^, MD; Francois Lejeune^14^, MD; Andre Ly^15^, MD; Michael Piagnerelli^16^, MD; Bertrand Sauneuf^17^, MD; Nicolas Serck^18^, MD; Thibaud Soumagne^19^, MD; Piotr Szychowiak^20,21,22^, MD; Julien Textoris^23,24^, MD; Benoit Vandenbunder^25^, MD; Christophe Vinsonneau^26^, MD; Jean-Baptiste Lascarrou^22,27^, MD for the COVADIS study group.

Table S1: Antiviral standard procedure in the participating centers

|  | Hydroxychloroquine | | Lopinavir/Ritonavir | | Remsedivir | |
| --- | --- | --- | --- | --- | --- | --- |
|  | Dose and duration | Rules for stopping | Dose and duration | Rules for stopping | Dose and duration | Rules for stopping |
| 1 | 400mg/day during 5 days | prolonged QTc, arythmia | NA | NA | NA | NA |
| 2 | 600mg/day during 10 days | prolonged QTc | 800mg/day during 6 days | GFR<30ml/min or ASAT/ALAT>5N | NA | NA |
| 3 | NA | NA | NA | NA | NA | NA |
| 4 | 400mg/day during 5 days | prolonged QTc | NA | NA | NA | NA |
| 5 | 800mg day 1 then 400mg during 9 days | prolonged QTc | 800mg/day during 14 days | GFR<30ml/min or ASAT/ALAT>5N | 200mg day 1 then 100mg during 9 days | GFR<30ml/min or ASAT/ALAT>5N |
| 6 | 400mg/day during 5 days | prolonged QTc | NA | NA | NA | NA |
| 7 | NA | NA | 800mg/day during 14 days | GFR<30ml/min or ASAT/ALAT>5N | NA | NA |
| 8 | 400mg/day during 5 days | prolonged QTc | NA | NA | 100mg/day during 7 days | None |
| 9 | 600mg/day during 10 days | prolonged QTc | NA | NA | NA | NA |
| 10 | 800mg day 1 then 400mg during 4 days | None | 800mg/day during 14 days | None | 200mg day 1 then 100mg during 9 days | None |
| 11 | 600mg/day during 10 days | prolonged QTc, RRT | 800mg/day during 14 days | GFR<30ml/min or ASAT/ALAT>5N | 200mg day 1 then 100mg during 9 days | None |
| 12 | 400mg/day during 5 days | ICU admission | NA | NA | NA | NA |
| 13 | 400mg/day during 5 days | prolonged QTc | 800mg/day during 10 days | None | 200mg day 1 then 100mg during 9 days | Needs for RRT |
| 14 | 400mg/day during 5 days | none | 800mg/day during 14 days | None | 200mg day 1 then 100mg during 9 days | None |
| 15 | 400mg/day during 5 days | prolonged QTc, arythmia | 800mg/day during 5 days | None | NA | NA |
| 16 | 600mg/day during 7 days | prolonged QTc, arythmia | 800mg/day during 7 days | Diarrhea,  GFR<30ml/min | NA | NA |
| 17 | 800mg day 1 then 400mg during 9 days | prolonged QTc | 800mg/day during 14 days | GFR<30ml/min or ASAT/ALAT>5N | 200mg day 1 then 100mg during 9 days | None |
| 18 | NA | NA | NA | NA | NA | NA |
| 19 | 800mg day 1 then 400mg during 5 days | arythmia | NA | NA | NA | NA |
| 20 | 400mg/day during 5 days | prolonged QTc | NA | NA | NA | NA |
| 21 | 600mg/day during 10 days | ICU admission | 800mg/day during 7 days* | None | NA | NA |

RRT: Renal Replacement Therapy

* Cease L/R administration after RCT publication (doi 10.1056/NEJMoa2001282)

**Table S2: Associated factors with ICU survival**

|  | Non survivors  N=166 | survivors  N=240 | *P value*^1^ |
| --- | --- | --- | --- |
| Age, mean±SD | 66 ±10 | 62 ±10 | <0.001 |
| Gender, men, n (%) | 131 (79) | 182 (76) | 0.55 |
| BMI, kg/m^2^, mean±SD | 29.5 ±5 | 29.9 ±5 | 0.39 |
| Hypertension, n (%) | 99 (60) | 130 (54) | 0.31 |
| Uncomplicated diabetis mellitus, n (%) | 33 (20) | 45 (19) | 0.80 |
| Complicated diabetis mellitus, n (%) | 22 (13) | 11 (5) | 0.003 |
| Chronic kidney disease, n (%) | 22 (13) | 10 (4) | 0.001 |
| Periphereal artery disease, n (%) | 14 (8) | 11 (5) | 0.14 |
| History of myocardial infarction, n (%) | 16 (10) | 20 (8) | 0.72 |
| Charlson Comorbidity index, median [IQR]  = 0  = 1  ≥ 2 | 1 (0-3)  58 (35)  34 (21)  74 (45) | 1 (0-2)  110 (46)  70 (29)  60 (25) | < 0.001  < 0.001 |
| PEEP (cmH_2_O), mean±SD | 11.5 ±3 | 11.5 ±3 | 0.99 |
| Plateau pressure (cmH_2_O), mean±SD  N=159/207 | 24.4 ±4 | 23.0 ±4 | 0.001 |
| P/F, mean±SD | 121 ±48 | 131 ±51 | 0.07 |
| NO, n (%) | 27 (16) | 24 (10) | 0.07 |
| Hydroxychloroquine, n (%) | 110 (66) | 148 (62) | 0.40 |
| Lopinavir/ritonavir, n (%) | 37 (22) | 48 (20) | 0.62 |
| Remdesivir, n (%) | 8 (5) | 13 (5) | 0.82 |
| Corticosteroids^2^, n (%)  N=221/171 | 41 (25) | 40 (18) | 0.10 |
| Macrolides | 106 (64) | 149 (62) | 0.76 |
| Co-infection | 23 (14) | 26 (11) | 0.36 |
| Day 14 Ventilatory mode  Death  Controlled or VV-ECMO  Pressure support  Extubated | 94 (57)  58 (35)  13 (8)  1 (1) | 0 (0)  80 (33)  73 (30)  87 (36) | <0.001 |
| Acute kidney injury, n (%)  - No  - Yes without RRT  - Yes with RRT | 45 (27)  72 (43)  49 (30) | 136 (57)  73 (30)  31 (13) | <0.001 |
| Peak Creatinine until Day 28, median [IQR]  n= 164/205 | 210 [112-429] | 103 [77-175] | < 0.001 |
| Cardiac injury, n (%) | 37 (22) | 18 (8) | < 0.001 |
| Pulmonary embolism, n (%) | 23 (14) | 35 (15) | 0.89 |
| Alive at D28, n (%) | 26 (16) | 240 (100) | < 0.001 |

AKI: acute kidney injury, see definition in the method section

BMI: body mass index; SD: standard deviation; P/F: PaO2/FiO2 ratio; NO: Inhaled nitric oxide;

^1^ P-value was calculated by Fisher exact test, t-test or Mann Whitney test as appropriate

^2^ some patients were included in a double blind RCT steroids vs placebo and are considered as missing data, most of the treated patients received steroids lately

Figure S1: Flow chart of the study


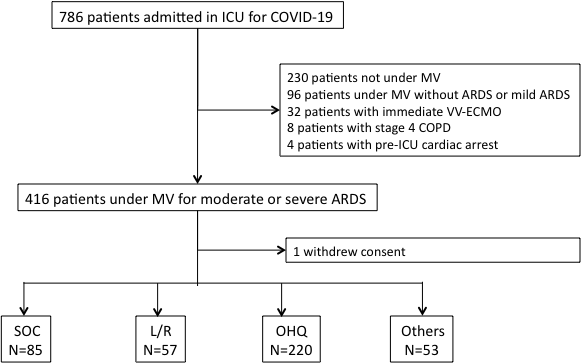


MV: mechanical ventilation, SOC: standard of care, L/R: Lopinavir/ritonavir, OHQ: hydroxychloroquine, Others: OHQ + L/R N= 32; OHQ + Remdesivir N= 10; Remdesivir N=11

Figure S2: Forest plot of antiviral strategies effect on ICU survival in a mixed multivariate model

OR (CI95%) were obtained through a generalized linear mixed model including antiviral strategies, age, sexe, Charlson comorbidity index, plateau pressure and P/F with centre as random effect. (N= 361). L/R: lopinavir/ritonavir ; OHQ: hydroxychloroquine.

Figure S3: Forest plot of variables associated with AKI in a mixed multivariate model


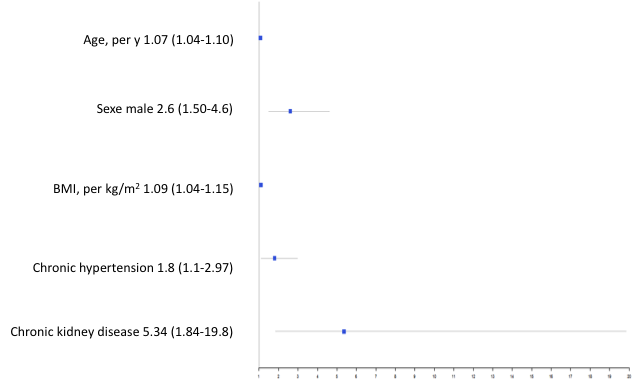
 OR (CI95%) were obtained through a generalized linear mixed model including lopinavir, hydroxychloroquine, age, sexe, BMI, chronic hypertension and moderate to severe chronic renal failure with centre as random effect. (N= 410). N= 410. AKI: acute kidney injury. Chronic kidney disease: moderate to severe chronic renal failure
